# Exploring Emotional Well‐Being in Exceptional Aging: Data from the SuperAging Research Initiative

**DOI:** 10.1002/alz70857_098016

**Published:** 2025-12-24

**Authors:** Claire M. Alexander, Kylie R. Kadey, Rhiana Schafer, Ozioma C. Okonkwo, Felicia C. Goldstein, Angela C. Roberts, Adam Martersteck, Amanda Cook Maher, Emily J Rogalski, The SuperAging Research Initiative

**Affiliations:** ^1^ Ann Arbor VA Healthcare System, Ann Arbor, MI, USA; ^2^ University of Michigan, Ann Arbor, MI, USA; ^3^ University of Chicago, Chicago, IL, USA; ^4^ University of Wisconsin, Madison, WI, USA; ^5^ Emory University School of Medicine, Atlanta, GA, USA; ^6^ Western University, London, ON, Canada

## Abstract

**Background:**

Emotional and social well‐being factors including purpose in life, emotional support, social engagement, and self‐efficacy predict better cognitive functioning among older adults. These factors may contribute to the extraordinary memory of “SuperAgers,” adults age 80+ with episodic memory at least comparable to 50‐60‐year‐olds. Previous single‐site SuperAging studies suggest stronger interpersonal relationships may contribute to the SuperAging phenotype. Building on initial findings, we examine emotional well‐being using the NIH Toolbox‐Emotion Battery (NIHTB‐EB) in SuperAgers compared to cognitively‐average peers (Controls).

**Method:**

Cross‐sectional cognitive, demographic, and NIHTB‐EB data were queried from the SuperAging Research Initiative, a multisite longitudinal cohort with harmonized multidisciplinary data collection. We examined NIHTB‐EB T‐scores between SuperAgers (*n* = 65) and Controls (*n* = 71), by sex, and within self‐reported socialized racial identity using independent samples *t*‐tests.

**Result:**

Most participants self‐identified as female (58.8%) and White (84.6%), with an average age of 85 (range=80‐100). Overall, SuperAgers did not differ from Controls on NIHTB‐EB measures, with both groups reporting trends toward lower anger, sadness, and perceived stress compared to the normative sample, and slightly higher general life satisfaction (effect sizes ∼0.5‐1 SD). Across the sample, males reported slightly higher availability of instrumental support (*p* = .003) and slightly lower affective fear (*p* = .046) compared to females. Within White participants, SuperAgers did not differ from Controls on NIHTB‐EB measures. However, within the small subset of Black/African‐American participants, SuperAgers reported lower affective fear compared to Controls (*p* = .036).

**Conclusion:**

These findings further characterize the emotional well‐being of cognitively healthy adults age 80+. Consistent with prior SuperAging reports, positive emotional well‐being was observed. Whereas prior research indicates older men tend to report higher social isolation and less social support compared to women, males in our study reported a higher perception of available resources from their social networks than females. Other studies have shown similarly contradictory findings, with men reporting more social ties but women reporting greater group involvement and emotional support. Within‐race comparisons require cautious interpretation given limited sample size; however, the reported lower affective fear in Black SuperAgers compared to Black Controls may reflect the impact of social determinants of health, life stressors, or allostatic load on cognitive aging.

